# The Songdo consensus: Development of minimum reporting standards for studies of intervention in idiopathic anal fistula using a modified nominal group technique

**DOI:** 10.1111/codi.17300

**Published:** 2025-01-23

**Authors:** Shivani Joshi, Luke Hanna, Dong Ho Cho, Pankaj Garg, Tamara Glyn, Brooke Gurland, Do‐Yeon Hwang, Kiduk Kim, Paulo Gustavo Kotze, Jong Kyun Lee, Amy L. Lightner, Klaus E. Matzel, Kapil Sahnan, Francis Seow‐Choen, Ali Shafik, Daeyoun Won, David D. E. Zimmerman, Phil J. Tozer

**Affiliations:** ^1^ Department of Surgery and Cancer Imperial College London London UK; ^2^ Robin Phillips' Fistula Research Unit St Mark's Hospital London UK; ^3^ Department of Coloproctology Seoul Songdo Hospital Seoul South Korea; ^4^ Garg Fistula Research Institute Panchkula India; ^5^ University of Otago Christchurch New Zealand; ^6^ Stanford University Stanford California USA; ^7^ Pontifícia Universidade Católica Do Paraná (PUCPR) Curitiba Brazil; ^8^ Scripps Clinic La Jolla California USA; ^9^ Scripps Research Institute La Jolla California USA; ^10^ Department of Coloproctology University Erlangen‐Nürnberg Erlangen Germany; ^11^ Seow‐Choen Colorectal Centre Singapore Singapore; ^12^ Kasr Al‐Aini Faculty of Medicine Cairo Egypt; ^13^ Elisabeth‐TweeSteden Hospital Tilburg Netherlands

**Keywords:** anal fistula, cryptoglandular anal fistula, idiopathic anal fistula, minimum reporting standards, nominal group technique

## Abstract

**Aim:**

Cryptoglandular anal fistulas carry a substantial burden to quality of life. Surgery is the only effective curative treatment but requires balancing fistula healing against pain, wounds and continence impairment. Sphincter‐preserving procedures do exist but demonstrate variable rates of success. A lack of consistency and precision in outcome reporting and methodological quality hinders effective evidence‐based decision‐making. We aimed to establish a series of minimum reporting standards for interventional studies in idiopathic anal fistula, to eradicate low‐quality studies, thus providing a consistent baseline of useful evidence.

**Methods:**

An international group of 16 experts participated in a modified nominal group technique consensus. The nominal question was: ‘What should be the minimum set of reporting standards for studies of intervention in idiopathic anal fistula?’ The process was conducted between May and June 2023, culminating in a hybrid in‐person/virtual meeting that took place at the Songdo International Proctology Symposium in June 2023.

**Results:**

Initial idea generation resulted in 37 statements within the first round. Themes included variable reporting of follow‐up and incontinence. Participants indicated their agreement via a 9‐point Likert scale. Any statement achieving >70% consensus was retained. Subsequent group discussion condensed the list to 11 statements for further voting and a final minimum set of 12 reporting standards was created.

**Conclusion:**

To date, this is the first study dedicated to developing minimum reporting standards for interventional studies in idiopathic anal fistula using a modified nominal group technique. These standards will instruct researchers in producing meticulous, high‐quality studies that are accurate, transparent and reproducible.

## INTRODUCTION

Poor‐quality reporting can lead to redundant research [1, 2], financial costs [1, 3, 4], a loss of scientific integrity and a risk of patient harm [1]. Minimum reporting standards can address this by ensuring transparent, meticulous reporting to provide reproducible and comparable results [5].

The symptoms of cryptoglandular anal fistula can be devastating for quality of life. Surgery, whilst the only effective curative treatment, risks pain, recurrent wounds and potential incontinence, which must be balanced with complete fistula healing. Sphincter‐preserving procedures exist but none carries a high success rate, and all demonstrate variable healing rates in published studies [6, 7]. The European Society of Coloproctology guidelines for cryptoglandular anal fistula provide valuable recommendations for management [8]; however, enhancing research quality remains crucial. Inconsistent outcome reporting and methodological quality are demonstrated in short and variable follow‐up, inconsistent definitions of healing, and inaccurate reporting of complications, for example recurrence and incontinence, impeding evidence‐based decision‐making [9].

A core outcome set (COS) for cryptoglandular anal fistula has been created [10] and a core outcome measurement set (COMS) is being built [11]. These determine the key outcomes relevant to patients and clinicians (such as quality of life), and the optimal measurement instruments for their assessment. However, other methodological problems relating to patient selection, follow‐up length, confusion about terms relating to healing, failure of repair and recurrence, and ambiguity in the methods of assessing outcomes like incontinence will not be completely addressed by the COS or COMS.

As a complementary exercise, the development of disease‐specific reporting standards will form part of a framework to avoid common pitfalls when designing and reporting studies.

The Songdo consensus addresses the P (Patient), I (Intervention) and C (Comparison) of the 'PICO' framework and adds nuance to the reporting of the Os (Outcomes) which were selected in the anal fistula COS, and which will have measurement instruments selected for them in the anal fistula COMS, according to COSMIN guidance.

A systematic review (Appendix S1) identified an absence of disease‐specific standards beyond generic guidelines, such as CONSORT [12] or STROBE [13]. This is important given the challenges in reporting perianal fistula studies, for example, numerous definitions for fistula healing and various timepoints for assessment [9], with no established consensus. Implementing these standards will ensure that sequelae, for example, healing, recurrence and incontinence, are reported with consistent granularity, facilitating accurate comparisons across studies.

The nominal group technique (NGT), established in 1975 [14], is widely utilised to achieve consensus in healthcare‐based research [15], by generating ideas/themes in response to a ‘nominal question’ [16]. ‘Nominal group’ refers to the importance of singular contributions within a group environment [17]. Several studies have used the method successfully to establish guidelines or transform management for a clinical condition [15, 16, 17, 18, 19]. The small group, discursive nature of the NGT allows for detailed, nuanced and specific discussion of narrow questions, which is difficult in a Delphi‐style consensus process.

Our objective was to develop minimum reporting standards for interventional studies of idiopathic anal fistula, using expert opinions and a modified NGT. These standards are intended to be applicable within the international scientific community, complementing the COS and COMS, which are developed according to very strict guidance, and despite which heterogeneity in patient selection, intervention and comparator description and some nuances around reporting of outcomes will remain neglected. Where overlap is perceived to exist, particularly in areas related to patient involvement, the COS and COMS supervene, although the design of the Songdo reporting standards was undertaken considering the COS and the need for a COMS, and they are expected to trend in parallel, with some shared authorship.

## METHODS

The NGT was chosen for its ability to stimulate complex thematic discussion within a structured approach, producing immediate quantitative and qualitative data in response to the ‘nominal question’ [17, 20, 21]. Participants must individually generate ideas prior to group discussion and subsequently rank ideas in order of preference, to produce a list of priorities [16, 17, 19].

**FIGURE 1:**
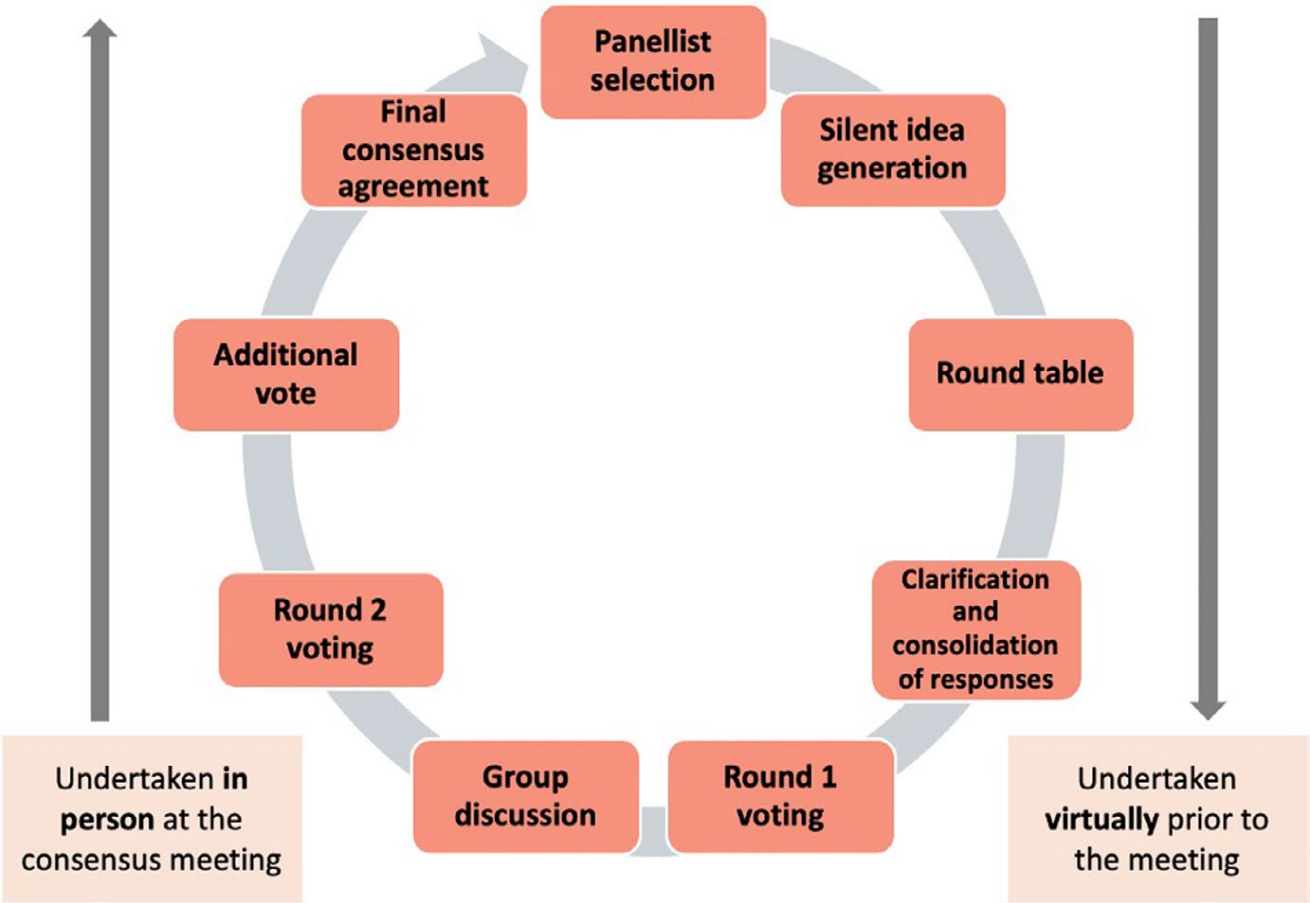
Modified nominal group technique.

The NGT was modified as demonstrated in Figure 1, such that the preliminary stages (idea generation, first voting round) were undertaken via email correspondence. The final stages (clarification through group discussion, second voting round) were scheduled for a consensus meeting at the Songdo International Proctology Symposium, Seoul, Korea, in June 2023.

### Panellist selection

The research leads were responsible for panellist selection, with centralised oversight of invitations. Panellists were selected based on expertise (high‐volume tertiary practice and/or number of publications, in addition to regular participation as peer reviewers in fistula studies) and geographical distribution.

Based on their expertise, attendees speaking at the symposium were invited to participate in the NGT. Further purposive sampling was undertaken To offset any lack of geographical variation, resulting in 16 international experts participating. A hybrid in‐person/virtual meeting was planned to allow an expert from each continent to contribute. Patients were not included in the panel due to the technical nature of the study, addressing questions of shared methodology, consistency and reproducibility.

### Drafting and review of items

An abstract detailing the consensus process was sent to all invited attendees. An explanation of reporting standards was provided to ensure participant understanding. The nominal question was ‘What should be the minimum standard of reporting for studies of intervention in idiopathic anal fistula?’

The initial phase consisted of independent idea generation, generating up to three responses to the posed question. This was based on established methodology within previous NGT studies [17], ensuring data collection and analysis remained focused and manageable.

Ideas were collated and clarified by the lead researchers, SJ and PT. Any similar responses were combined. Using a systematic approach reduced the potential for bias and power dynamics to impact the outcome. [16].

For this study, it was determined that thematic cataloguing of items and voting to create a list of statements of equal priority would be more pertinent than ranking ideas in order of preference to create a prioritised list, as originally described in the NGT.

### Achieving consensus

The first voting round was conducted online prior to the consensus meeting. Participants voted on statements using a 9‐point Likert scale (where 7–9 represented agreement and 1–3 represented disagreement). The lead researcher collated and analysed the results. Items that did not achieve 70% consensus were discarded.

The subsequent stages were conducted during the hybrid in‐person/virtual consensus meeting in Seoul. All statements, including retained and discarded statements, were presented to the panel. Participants were given an opportunity to discuss and clarify the meaning and scope of each statement. Any overlapping or superfluous statements were merged or discarded.

The group discussion produced a list of preliminary statements for the second voting round. Participants voted on each statement, indicating agreement or disagreement according to a 9‐point Likert scale. Any statement not achieving 70% agreement was removed. Further discussion and an additional re‐vote were required for any statement with a discrepancy or disagreement.

The consensus meeting was recorded, transcribed verbatim and thematically analysed by a trained qualitative researcher to systematically identify and organise key themes for the final manuscript.

## RESULTS

**FIGURE 2:**
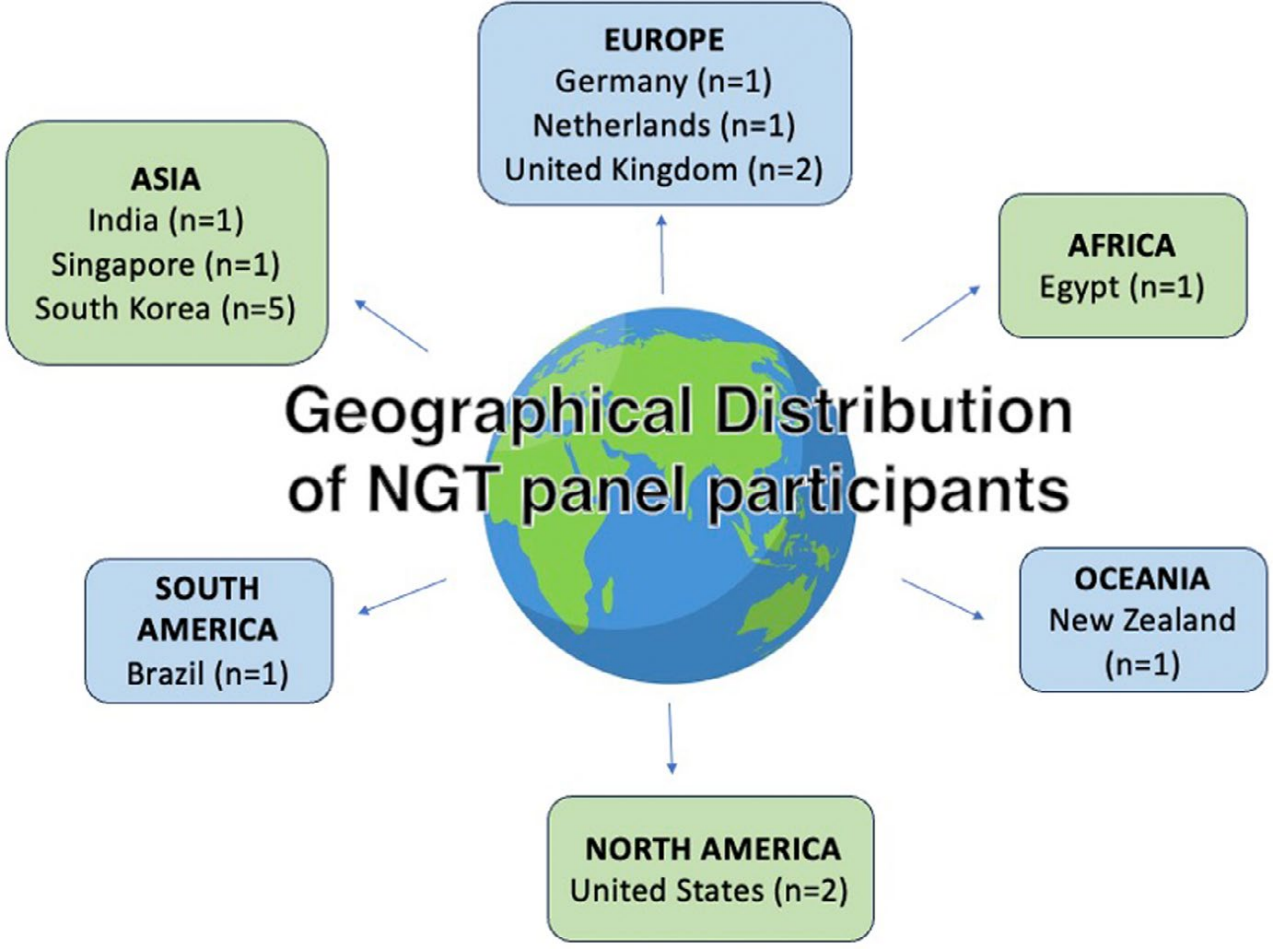
Geographical distribution of NGT panel participants per continent: Brazil (*n* = 1), Egypt (*n* = 1), Germany (*n* = 1), India (*n* = 1), Netherlands (*n* = 1) New Zealand (*n* = 1), Singapore (*n* = 1), South Korea (*n* = 5), UK (*n* = 2), United States (*n* = 2). Overall gender distribution 14 males/2 females. NGT, nominal group technique.

The NGT panel was composed of 16 experts within perianal fistula research, from varying geographical locations (Figure 2).

Ideas were generated in response to the nominal question under the following thematic subheadings. Similar ideas were combined and catalogued under these headings.

1. How should the follow‐up duration for reporting an outcome be described?
2. How should faecal incontinence be described?
3. How should patient cohorts be selected and identified?
4. How should success be described?
5. How should the interventions (including comparators) be described?

The results of the first voting round are demonstrated in Appendix S2. All statements scoring above 70% agreement were retained for further discussion and adaptation. The panel were presented with the retained and discarded statements. After further discussion, similar statements were amalgamated, and any ambiguous statements were clarified and modified accordingly.

The second voting round was structurally identical to the first round. The results are presented in Appendix S3. Both rounds had a 100% response rate.

Despite reaching consensus threshold, the statement ‘Follow‐up should be patient and healthcare professional delivered’ initiated further discussion from two members of the panel. In view of this, an additional vote was undertaken (Appendix S3), incorporating the original statement but also two modified versions of the same statement, produced through iterative discussion. Any statement achieving greater than 70% was ‘consensus in’. This led to the inclusion of the original statement in addition to the statement ‘Follow‐up should be reported as patient or healthcare professional delivered’.

**FIGURE 3:**
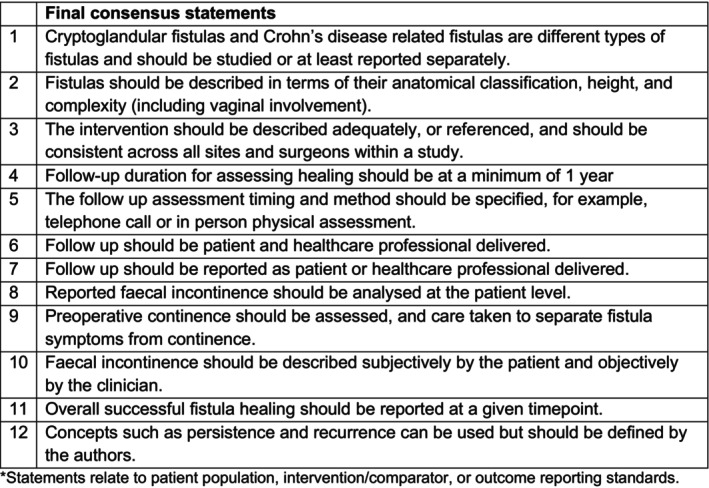
Final list of minimum reporting standards in studies of intervention in idiopathic anal fistula.

The consensus process resulted in 12 minimum reporting standards (Figure 3).

## DISCUSSION

Inconsistent reporting hinders meaningful comparisons and effective meta‐analyses [9, 22]. Establishing reporting standards will enhance the scientific basis of studies and expand their clinical utility [23]. Herein, this consensus process yielded 12 reporting standards that should be adhered to when reporting an interventional study of idiopathic anal fistula.

Whereas a COS selects the outcomes to be measured, and a COMS identifies the optimal measurement instruments to do so, a minimum reporting standard addresses all aspects of a study, supporting fair, meaningful, consistent and reproducible reporting. Although generic reporting standards exist, the specific and focused reporting standards developed by a global team of fistula experts, researchers and reviewers underlines the value of this specific set of standards.

The panel recommended that ‘Fistulas should be described in terms of their anatomical classification, height and complexity (including vaginal involvement)’. It was noted that the ideal anatomical classification does not yet exist or is not fully recognised, particularly regarding fistula height. Likewise, it was recognised that defining complexity in studies can be difficult but accepted that complexity should be described as a minimum.

Furthermore, ‘The intervention should be described adequately, or referenced, and should be consistent across all sites and surgeons within a study’. It was accepted that technical variations will always occur, and that many ‘standard’ operations are already done in slightly different ways by different surgeons. For that reason, a full description of the technical steps was advised at all times.

The panel recommended ‘Follow‐up duration for assessing healing should be at a minimum of 1 year’. An unpublished analysis of the Seoul National Database (*n* = 1300) was referenced, in which the median time to recurrence was 7.7 months (range 1.4–36.1, with a standard deviation of 6.8), supporting the hypothesis that most recurrences are likely to have occurred within 1 year, although not all are apparent immediately.

This accounts for more extensive wounds that may take longer to heal and recognises that a median follow‐up of 1 year would, by definition, include half the total number of patients with follow‐up length shorter than 1 year. The COMS (in progress) will address the measurement instruments to be used, including for determining fistula healing and follow‐up duration for each outcome, but this reporting standard can add to that by ensuring a minimum 1‐year follow‐up as standard practice. Authors should include only patients who have completed a full year of follow‐up, avoiding the inclusion of patients who have not.

The group recommended ‘Follow‐up should be patient and healthcare professional delivered’, emphasising the importance of including both patient‐reported and clinician‐reported outcomes within follow‐up consultations, rather than relying solely on clinician‐reported data. This approach captures objective and subjective data for a comprehensive assessment of patient progress after an intervention.

Similarly, the reporting standards highlight that ‘Reported faecal incontinence should be analysed at the patient level’. This refers to assessing individual patient outcomes, before the aggregation of data, to prevent generalisations that may obscure meaningful details. By evaluating continence for each patient individually, we can better understand treatment effects and later combine the data to produce reliable conclusions about the impact of an intervention. For example, whichever measurement instrument is chosen by the COMS would assess the preoperative and postoperative continence in each individual patient. These data can then be aggregated to describe the number of patients in whom a deterioration in continence is seen (e.g., ‘*X* patients developed a significant deterioration in their continence score after intervention’, rather than ‘the average difference in continence score across the patients after intervention was *X* points’).

The group recommended ‘Preoperative continence should be assessed, and care taken to separate fistula symptoms from continence’. This is exemplified in the inaccurate use of (sometimes averaged) scores of validated indices. Importantly, for example, a preoperative incontinence score may be erroneously high due to pad usage from fistula discharge. The score subsequently may not change postoperatively despite new minor incontinence. The actual reduction in continence post‐intervention is lost as the new continence impairment is balanced by the fistula‐driven symptoms from before intervention. This problem is amplified when averaged cohort‐level analysis of continence scores is used, as deterioration in continence in some patients is diluted amongst those in whom the score does not change (or even goes up). This results in an average score that is unrepresentative of most individuals in a mixed group with two broad cohorts (those in whom the continence changes and those in whom it does not). Instead, changes should be measured at a patient level, and described in the same way, so that the size of the group in whom a deterioration occurs and the extent of deterioration of continence in patients in that group can easily be identified.

The group recommended ‘Overall successful fistula healing should be reported at a given timepoint’, acknowledging that persistence, recurrence and initial healing are different concepts that carry importance to some researchers. However, they concluded that the overarching question a study must answer is the number of patients who still have a fistula after a specific timepoint. Describing initial healing, persistence and recurrence is confusing if, for example, ‘overall healing at 1 year’ is not also described.

The group noted that a new fistula with a new morphological appearance is often incorrectly defined in studies as a recurrence. It was therefore decided that ‘Concepts such as persistence and recurrence can be used but should be defined by the authors’.

Finally, the group decided not to include a recommendation based around imaging modalities, acknowledging that some centres may be limited in their resources, for example advanced imaging modalities, and therefore emphasised that these centres should not be disqualified from reporting due to a lack of resources. The group stressed the importance of smaller studies, which may still carry value, particularly if there is a specific reason for the study, for example technical variation, marketing, geographical significance or rare situations/fistula anatomy.

Overall, these minimum reporting standards offer distinct, disease‐specific recommendations that scope beyond existing reporting guidelines, and address separate questions from those covered by a COS and COMS. These recommendations are tailored for idiopathic anal fistula, addressing key aspects that are not covered in existing generic reporting guidelines, such as consistent reporting of follow‐up, incontinence and the importance of distinguishing between different fistula types within studies. They will ensure that the subtle nuances inherent within the study of idiopathic anal fistula are sufficiently reported, resulting in greater precision when assessing the outcomes and benefits of a study.

There were several benefits of using a modified NGT. It is a structured, focused method for idea generation [16], permitting non‐hierarchical contributions and thereby reducing the effect of dominant personalities and the impact of ‘groupthink’ [17, 24]. The process is flexible, swift and cost‐effective, providing immediate quantitative and qualitative results that can easily be analysed after a single meeting [17, 20, 25], which is particularly important in the context of an international symposium. The technique can give rise to a broad range of ideas, in comparison to other group methodologies [17].

However, the technique does have limitations. First, the group makeup may limit the general applicability of any findings [24, 26]. We addressed this by including a participant from each continent, although some continents were better represented. Additionally, the validity of results may be affected by small numbers, allowing a single vote to possibly skew the results [24, 27]. Some participants may find the structured framework of the approach restrictive [24, 28]. This was managed by skilled facilitators, with previous experience in using the NGT to achieve an unbiased consensus, and by using a group of highly experienced participants used to creating and reviewing research in this disease area.

Finally, we acknowledge that there may be slight overlap between the minimum reporting standards developed by this group and the development of a COMS [11] which is currently in progress. Whilst these reporting standards are largely complementary to the COMS, the COMS should supersede the standards where overlapping advice occurs, particularly where it pertains to patient choice.

In practice, both initiatives are distinct yet synergistic concepts that can be used to improve the quality of research that is undertaken for idiopathic fistulising disease. Minimum reporting standards are technical standards which provide baseline reporting requirements for study design and the nuances associated with reporting specific outcomes, such as incontinence. In contrast, the COMS is a patient‐centred framework which focuses on providing detailed guidance on which patient‐approved outcome measures should be utilised within clinical studies and at which timepoints. The combined use of these initiatives will provide researchers with comprehensive, robust guidance across the various aspects of study design and reporting, effectively bridging any gaps in quality. However, we emphasise the COS and COMS should take precedence when reporting outcomes of a study, whereas these reporting standards add nuance and detail around areas which experts have identified as specific and recurrent problems in fistula research outside of these areas.

We have established international minimum reporting standards for interventional studies in idiopathic anal fistula, using a modified NGT. We contend that the NGT can be successfully modified to result in dynamic and fruitful conversation to produce reporting standards that will truly enhance clinical research within the field of perianal fistula surgery.

## AUTHOR CONTRIBUTIONS

**Shivani Joshi:** Conceptualization; methodology; data curation; investigation; formal analysis; software; project administration; writing – original draft; visualization; validation. **Luke Hanna:** Data curation; writing – review and editing; project administration; investigation. **Dong Ho Cho:** Writing – review and editing; investigation. **Pankaj Garg:** Writing – review and editing; investigation. **Ms Tamara Glyn:** Writing – review and editing; investigation. **Brooke Gurland:** Writing – review and editing; investigation. **Do‐Yeon Hwang:** Investigation; writing – review and editing. **Kiduk Kim:** Writing – review and editing; investigation. **Paulo Gustavo Kotze:** Investigation; writing – review and editing. **Jong Kyun Lee:** Writing – review and editing; investigation. **Amy L. Lightner:** Conceptualization; methodology; data curation; supervision; resources; writing – review and editing; investigation. **Klaus E. Matzel:** Writing – review and editing; investigation. **Kapil Sahnan:** Writing – review and editing; investigation. **Francis Seow‐Choen:** Writing – review and editing; investigation. **Ali Shafik:** Writing – review and editing; investigation. **Daeyoun Won:** Writing – review and editing; investigation. **David D. E. Zimmerman:** Writing – review and editing; methodology; conceptualization; data curation; supervision; resources; investigation. **Phil J. Tozer:** Conceptualization; methodology; data curation; supervision; visualization; resources; writing – review and editing; formal analysis; investigation; validation.

## FUNDING INFORMATION

There is no grant support or funding to declare for this article. A funder did not play a role in the consensus process or influence the decisions reached.

## CONFLICT OF INTEREST STATEMENT

Professor Amy Lightner is a consultant for Boomerang Medical. Mr Phil Tozer has acted as speaker and/or served on advisory boards for Takeda Falk and Ferring. All other authors declared no competing interests.

## ETHICS STATEMENT

This study did not require ethical approval.

## CONSENT

No patients were involved in this study.

## Supporting information


Appendix S1:

Appendix S2:

Appendix S3:

## Data Availability

We have included a document of supplementary materials alongside the manuscript for submission. Given the nature of the project, no advanced data analysis was conducted as part of this work. Members of the public can contact the corresponding author regarding enquiries relating to the project's consensus process; however, key information is included in the main article and online supplementary materials.
