# Supplementary material for: The Songdo consensus: Development of minimum reporting standards for studies of intervention in idiopathic anal fistula using a modified nominal group technique
Source: Colorectal Dis. 2025 Jan 23;27(1):e17300. doi: 10.1111/codi.17300 (PMC11758350; doi:10.1111/codi.17300)
Supplement: Supplementary file 1 — Appendix S1: [file CODI-27-0-s001.docx]

**Systematic review of minimum reporting standards for studies of intervention in idiopathic anal fistula**

**Search strategy**

**Pubmed (January 1950 to August 2024)**

(Cryptoglandular fistula OR cryptoglandular anal fistula OR idiopathic anal fistula OR perianal fistula) AND (minimum reporting standards OR reporting standards OR reporting guidelines OR consensus guidelines)

**Embase Classic+Embase (January 1950 to August 2024)**

1 exp anus fistula/ or cryptoglandular fistula.mp. or exp rectum fistula

2 cryptoglandular anal fistula.mp.

3 idiopathic anal fistula.mp.

4 perianal fistula.mp. or anus fistula/

5 1 or 2 or 3 or 4

6 exp practice guideline/ or minimum reporting standards.mp.

7 reporting standards.mp.

8 reporting guidelines.mp.

9 consensus guidelines.mp. or exp consensus/

10 6 or 7 or 8 or 9

11 5 and 10

**Ovid MEDLINE(R) ALL (January 1950 to August 2024)**

1 exp Rectal Fistula/ or Cryptoglandular fistula.mp.

2 cryptoglandular anal fistula.mp.

3 idiopathic anal fistula.mp.

4 perianal fistula.mp.

5 1 or 2 or 3 or 4

6 minimum reporting standards.mp.

7 reporting standards.mp.

8 exp Guidelines as Topic/ or reporting guidelines.mp.

9 consensus guidelines.mp.

10 6 or 7 or 8 or 9

11 5 and 10

**PRISMA diagram:**

**
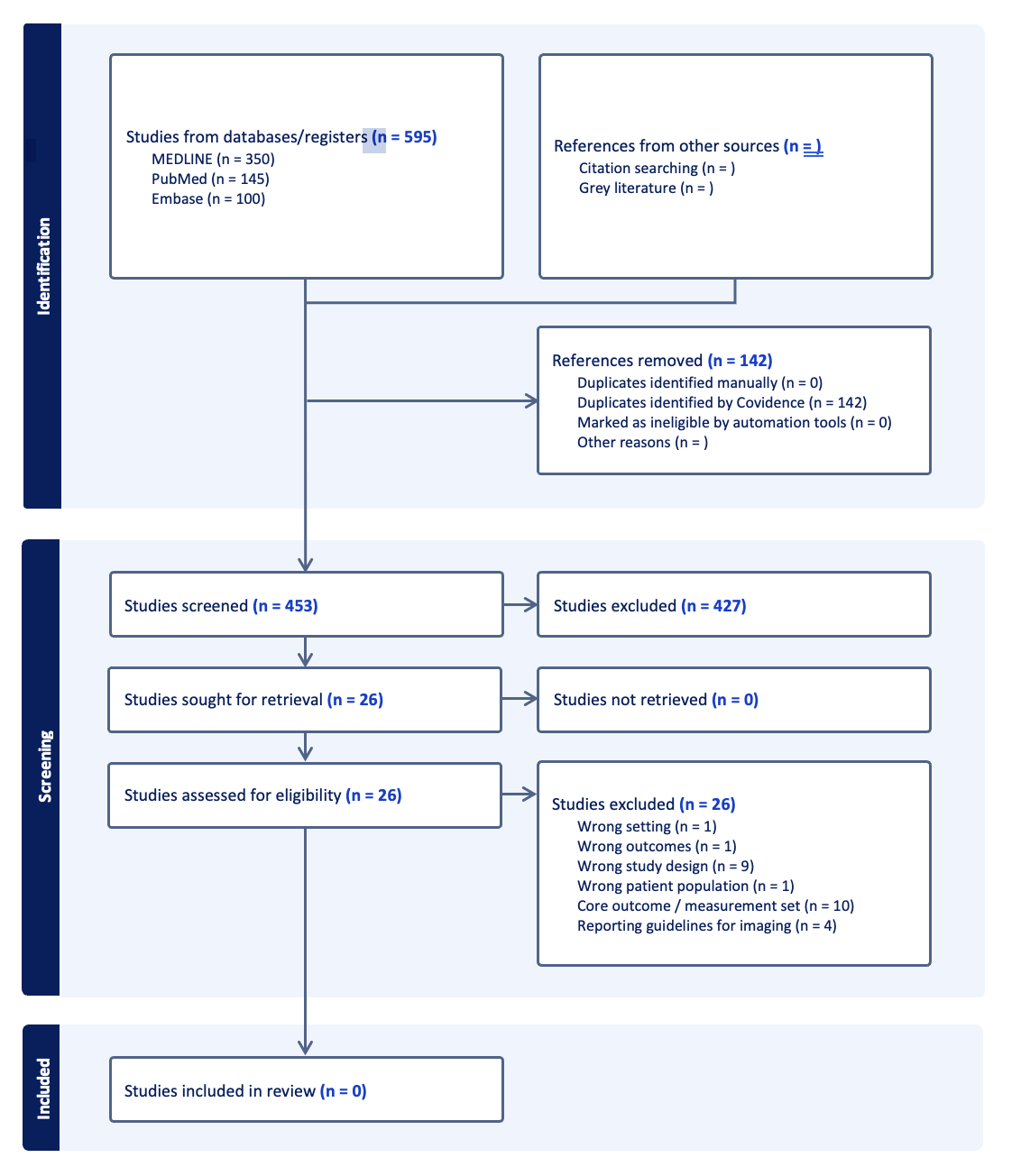
**
