# Supplementary material for: The Songdo consensus: Development of minimum reporting standards for studies of intervention in idiopathic anal fistula using a modified nominal group technique
Source: Colorectal Dis. 2025 Jan 23;27(1):e17300. doi: 10.1111/codi.17300 (PMC11758350; doi:10.1111/codi.17300)
Supplement: Supplementary file 2 — Appendix S2: [file CODI-27-0-s003.docx]

*Table 1a: How should the follow-up duration for reporting an outcome be described?*

| % | **How should the follow-up duration for reporting an outcome be described?** |
| --- | --- |
| 38.9 | Either with mean or median, at investigator’s discretion |
| 65.6 | Follow-up at 3-6 months and one year |
| 48.9 | Median follow-up at 12 months or less if appropriate |
| 76.7 | Follow-up assessment method should be specified, for example, telephone call, in person etc. |
| 74.4 | Follow-up can be different (timing and method of assessment) depending on the outcome. For example, incontinence can be measured remotely and earlier, whereas healing needs clinical assessment and should be later. |
| 71.1 | Follow up duration should be reported as a median (range). |
| 56.7 | Follow up duration from two weeks to 52 weeks. |
| 75.6 | Follow up should have a minimum duration of one year. |
| 70 | For healing, follow up should have a minimum duration (of 1 year) and then median + IQR. For incontinence, there should be a minimum follow-up of 3 months. |

*Table 1b: How should faecal incontinence be described?*

| % | **How should faecal incontinence be described?** |
| --- | --- |
| 68.9 | Faecal incontinence should be reported at a patient level. |
| 74.4 | Faecal incontinence should be reported at a patient level and with a score that includes urgency. |
| 62.2 | Faecal incontinence should be reported at a patient level, not as an average of scores. |
| 63.3 | Faecal incontinence should be reported as per the incidence of associated functional sequelae. |
| 73.3 | Preoperative continence should be assessed, and care taken to separate fistula symptoms from continence. |
| 64.4 | Continence should not only be reported in means or averages of scores; deterioration (and/or improvement) of continence should also be described in general terms (Parks or simply Major, Minor etc.) |
| 74.4 | Faecal incontinence should be described using validated patient reported outcomes. |
| 74.4 | Faecal incontinence should be described using a validated score. |
| 76.2 | Faecal incontinence should be described using the Wexner incontinence score |

*Table 1c: How should patient cohorts be selected and identified?*

| **%** | **How should patient cohorts be selected and identified?** |
| --- | --- |
| 86.7 | Cryptoglandular fistulas and Crohn’s disease-related fistulas are different types of fistulas and should be studied or at least reported separately. |
| 65.6 | For the description of Crohn’s related fistulas, the complex versus simple classification recommended by the American Gastroenterological Association (AGA) is the preferred approach. |
| 67.8 | Anatomic classifications can be utilised in the description of Crohn’s related fistulas. |
| 72.2 | Anatomic classifications, such as the Parks classification are the preferred approach when describing cryptoglandular fistulas. |
| 85.6 | Patient cohorts should be reported by aetiology (cryptoglandular/obstetric/IBD etc). |
| 72.2 | Patient cohorts should be reported by fistula height (where relevant), for example, lay open, fistulectomy and primary sphincteroplasty, ligation of intersphincteric tract etc. |
| 75.6 | Patient cohorts should be reported by complexity (where relevant). The inclusion of fistula of different complexity may be unavoidable but must then be stratified. |
| 74.4 | The numbers in the series should be proportional to the available data and prevalence of a specific disease or operation. If for example, 2000 flaps have been described, a series of 12 is no longer relevant. |
| 36.7 | Patient cohorts should be selected mixed. |
| 76.7 | Patient cohorts should specify Crohn’s versus non-Crohn’s patients. |
| 78.9 | Patient cohorts should specify vaginal involvement versus non-vaginal involvement. |
| 72.2 | Anatomic classification should be reported including ‘simple/complex’ and relationship of tract(s) and collections to the sphincter. Outcomes should be reported for each homogenous anatomical group. |

*Table 1d: How should success be described?*

| % | **How should success be described?** |
| --- | --- |
| 72.2 | Persistence and recurrence should be reported separately. |
| 70 | Persistence and recurrence should be reported together as failure at a given timepoint. |
| 70 | Initial healing and recurrence should be reported separately. |
| 51.1 | Initial healing and recurrence should not be reported separately but should be described as overall success at a given timepoint. |

*Table 1e: How should the interventions (including comparators) be described?*

| % | **How should the interventions (including comparators) be described?** |
| --- | --- |
| 83.3 | The interventions should be described in full. |
| 72.2 | The intervention should be consistent across all patients (regardless of surgeon/institution) within a given study. |
| 77.8 | In a multi-site study, a consistent surgical approach should be used and if necessary, taught, or at least a standard approach referenced. |
