# Supplementary material for: The Songdo consensus: Development of minimum reporting standards for studies of intervention in idiopathic anal fistula using a modified nominal group technique
Source: Colorectal Dis. 2025 Jan 23;27(1):e17300. doi: 10.1111/codi.17300 (PMC11758350; doi:10.1111/codi.17300)
Supplement: Supplementary file 3 — Appendix S3: [file CODI-27-0-s002.docx]

*Table 2: Round 2 Vote*

| % | **Round 2 Consensus Vote** |
| --- | --- |
| 93 | Follow-up duration for assessing healing should be at a minimum of 1 year |
| 87 | The follow up assessment timing and method should be specified, for example, telephone call or in person physical assessment. |
| 86.7 | Follow up should be patient and healthcare professional delivered. |
| 86.7 | Faecal incontinence should be reported at a patient level. |
| 93 | Preoperative continence should be assessed, and care taken to separate fistula symptoms from continence. |
| 86.7 | Faecal incontinence should be described subjectively by the patient and objectively by the clinician. |
| 93 | Cryptoglandular fistulas and Crohn’s disease related fistulas are different types of fistulas and should be studied or at least reported separately. |
| 93 | Fistulas should be described in terms of their anatomical classification, height, and complexity (including vaginal involvement). |
| 80 | Overall success should be reported at a given timepoint. |
| 86.7 | Concepts such as persistence and recurrence can be used but should be defined by the authors. |
| 86.7 | The intervention should be described adequately, or referenced, and should be consistent across all sites and surgeons within a study. |

*Table 3: Additional vote – ‘Follow up should be patient and healthcare professional delivered’*

| % | **Additional vote** |
| --- | --- |
| 84.7 | Follow-up should be patient and healthcare professional delivered. |
| 53.8 | Follow-up should be patient or healthcare professional delivered. |
| 84.6 | Follow-up should be reported as patient or healthcare professional delivered. |
